# Supplementary material for: Production of forest seedlings using sewage sludge and automated irrigation with ozonated cattle wastewater
Source: PLoS One. 2022 Oct 31;17(10):e0276633. doi: 10.1371/journal.pone.0276633 (PMC9621441; doi:10.1371/journal.pone.0276633)
Supplement: Attachment — Submitted filename: Response to Reviewer.docx [file pone.0276633.s002.docx]

Seropédica, Brazil, September 08, 2022.

To the Editorial Board of *PLOS ONE*

Dear Editor,

We would like to thank you for your message and for the reviewer’s comments on our manuscript (PONE-D-22-17230), entitled “Production of forest seedlings using sewage sludge and ozonated cattle wastewater: a potential for circular economy”.

We have modified the manuscript in response to the relevant and constructive reviewer comments. We highlight the modifications referring to the comments of the Reviewer 1 in red, and the Reviewer 2 in blue, and in green we present some modifications that, although they were not mentioned by the reviewers, also contributed to the improvement of the text, according to our judgment. It is worth mentioning that due to one of the comments by Reviewer #2, the title of the article was changed. We hope the revised version is now suitable for publication, however we are available for any clarifications.

In the sequence we present the Responses to each point raised by the academic editor and reviewer.

Best regards,

Daniel Fonseca de Carvalho (Corresponding author)

**Journal Requirements**

*1. Please ensure that your manuscript meets PLOS ONE's style requirements, including those for file naming.*

Response: all the format, style and names are checked.

*2. In your Methods section, please provide additional information regarding the permits you obtained to collect samples for the present study. Please ensure you have included the full name of the authority that approved the field site access and, if no permits were required, a brief statement explaining why.*

Response: Thanks for the comment. The experiment was carried out at a public university, where some authors are professors and others are undergraduate and graduate students. Therefore, there was no need for formal authorization to carry out this study. This information was included in lines 128-129.

*3. In your Data Availability statement, you have not specified where the minimal data set underlying the results described in your manuscript can be found. PLOS defines a study's minimal data set as the underlying data used to reach the conclusions drawn in the manuscript and any additional data required to replicate the reported study findings in their entirety. All PLOS journals require that the minimal data set be made fully available. For more information about our data policy, please see http://journals.plos.org/plosone/s/data-availability.*

*Upon re-submitting your revised manuscript, please upload your study’s minimal underlying data set as either Supporting Information files or to a stable, public repository and include the relevant URLs, DOIs, or accession numbers within your revised cover letter. For a list of acceptable repositories, please see http://journals.plos.org/plosone/s/data-availability#loc-recommended-repositories. Any potentially identifying patient information must be fully anonymized.*

Response: The main data collected in the field experiment were made available in the journal's system.

*4. Please include a copy of Table 2 which you refer to in your text on page 14.*

Response: Indeed, at the time of submission, the numbering of the tables was changed due to text links. This has been fixed in the new version.

**Responses to the reviewer’s comments**

Reviewer #1

*1. References are not well cited. Kindly update those; for example in line 80.*

Response: All references have been checked and adapted to the journal's rules. Thanks.

*2. Introduction is not clear, i hope that they will make investigate the current methods' advantages disadvantages and finally propose their research work. Line 110-112, you have proposed your work and why its necessary?*

Response: Changes have been made to the text in the paragraph citing the benefits of ozonation (lines 82-29).

*3. Kindly make a comparison of currently available methods and compare your research results with them.*

Response: In lines 79-81, we have mentioned the main methodologies for secondary treatment using advanced oxidation processes, recommended for organic effluents. However, in this study we used the ozonation methodology and no comparisons were made between CWW treatment methodologies.

Reviewer #2: The study represents the application of sewage sludge and cattle wastewater (CWW) to produce forest seedlings, attempting to reduce the release of waste and effluents into the environment. Generally, the results/findings are fine but some points should be considered before acceptance:

*1- The title includes the expression “Circular Economy”; however, it disappeared throughout the manuscript!! Why?*

Response: You are right: we included this expression because it represents the logic of our study, by reusing waste and cycling nutrients that would be lost and potentially polluting if disposed of incorrectly. However, although our technique fits the principles of the circular economy, we did not emphasize this in the text and chose to change the title.

*2- Do not repeat the words in the title to the “keywords”.*

Response: with the new title, the keywords were changed and we chose to add the term "circular economy".

*3- In the Introduction part, give some examples from the literature for the application of sewage/waste for forest seedlings.*

Response: We did not find in the literature studies that used cattle wastewater in the production of forest seedlings. However, studies carried out with sewage sludge were cited in lines 97 to 99.

*4- The last paragraph of Introduction should include the study objectives/procedures in brief.*

Response: The text referring to the objective of the study is presented in lines 117 to 119.

*5- The study should unveil the major gaps within the existing knowledge of the sewage/waste utilization for forest seedlings.*

Response: As we commented in one of the answers, the use of sewage sludge for the production of seedlings of tree species has increased in Brazil, but in none of the studies that have already been carried out has been used cattle water. Thus, we understand that this is a great contribution of our article.

*6- Are there any results for control run, without using sewage/waste?*

Response: thanks for your comment. Research groups from our University, in the forestry area, have been working with sewage sludge for many years, but without quantifying the volume of water applied in the production of forest seedlings. We started studying the water requirements of seedlings in 2018, maintaining the theme of using sewage sludge as a substrate for seedlings. Therefore, we did not intend to evaluate the growth of seedlings in another type of substrate. We intend to make this comparison in the next experiments. Thanks.

*7- The results should be enriched with statistical explanations.*

Response: Although no results were found for the production of seedlings with CWW, the results of the study were compared with those obtained using water from the T1 treatment (lines 339-343; 368-369; 396-397; 401-404; 437-439).

*8- The abstract and conclusion sections should be improved to show the main research findings.*

Response: the text has been changed.
